# Temperature-Sensitive Lipids Reveal Intraspecific Diversity in Bacteria Isolated from an Ancient Antarctic Microbial Mat

**DOI:** 10.1007/s00248-025-02583-4

**Published:** 2025-07-31

**Authors:** María Ángeles Lezcano, Daniel Carrizo, Miguel Ángel Lominchar, Laura Sánchez-García, Antonio Quesada, Víctor Parro

**Affiliations:** 1 Centro de Astrobiología (CAB), CSIC-INTA, 28850 Torrejón de Ardoz, Madrid Spain; 2 IMDEA Water Institute, Avenida Punto Com 2, 28805 Alcalá de Henares, Madrid Spain; 3 Departamento de Biología, C. Darwin 2, 28049, Universidad Autónoma de Madrid, Madrid, Spain

**Keywords:** Polar ecology, Antarctica, Psychrophiles, Psychrotrophs, Cold adaptation, Lipid biomarkers

## Abstract

Ancient Antarctic microbial mats harbour viable bacteria that have been exposed to extreme cold and arid conditions for hundreds of years. To delve into the molecular mechanisms underlaying their survival, we isolated 12 bacterial strains from a 1,000-year-old desiccated microbial mat from the McMurdo Ice Shelf and studied their lipid composition as a function of temperature. Six of the bacterial strains were classified as *Paenisporosarcina macmurdoensis*, and the other six as *Arthrobacter* sp., according to 16S rRNA gene analyses. Two strains of each taxon were incubated at their minimum and maximum growth temperatures, and the changes in their lipid profiles were analyzed. The proportion of major fatty acids (saturated, unsaturated, and *iso*- and *anteiso*-) remained relatively constant across temperature in the four strains, but the composition of hydrocarbons and alcohols differed between taxa (e.g., presence of unsaturated alkenes in *Arthrobacter* sp., or unidentified isoprenoid alcohols in *P. macmurdoensis*). This highlights the diagnostic value of non-fatty acid lipids and revealed a taxon-dependent lipid composition. Despite the taxon-associated lipid profile, incubation temperature also influenced lipid composition in both taxa, with higher temperature correlating with greater lipid richness. Interestingly, the two *P. macmurdoensis* strains showed distinct lipid profiles at 20°C, suggesting that intraspecific lipid diversity reflects within-species physiological variability with potential relevance for adaptation to temperature fluctuations in the mat. Therefore, assessing the influence of temperature on bacterial lipids is crucial for understanding their adaptation and survival in extreme environments, as well as for expanding species lipid inventories for biological interpretations of ancient samples.

**Supplementary Information:**

The online version contains supplementary material available at 10.1007/s00248-025-02583-4.

## Introduction

Extreme cold environments, such as Antarctica, are colonized by microbial life adapted (psychrophilic) or tolerant (psychrotolerant or psychrotrophic) to extremely low temperatures [1] that can also cope with low nutrient concentrations, dryness, freeze-thaw cycles and high UV radiation [2]. The McMurdo Ice Shelf, in Antarctica, is a large platform of floating ice in the ocean and an extremely cold location on Earth. Despite the extreme cold, the ice shelf has seasonal meltwater ponds on its surface [3, 4] that are colonized by benthic microbial mats [5] that undergo freeze-thaw and can eventually remain dry on the shores for hundreds of years [6]. Similarly, the McMurdo Dry Valleys, the largest area of ice-free ground in Antarctica and one of the coldest and driest places on Earth [7], has desiccated microbial mats that are thousands of years old on the shores of meltwater ponds [8–10]. Although these microbial mats have remained dry for hundreds or thousands of years, some microbial inhabitants have shown viability in laboratory conditions [6, 11], being key to understanding survival mechanisms under aridity and extremely low temperatures.

Rapid cell desiccation (i.e., anhydrobiosis) is one of the most relevant mechanisms that allows microorganisms to tolerate low temperatures and then resume metabolic activity when conditions are favourable [12, 13]. Most desiccation experiments have been conducted with microorganisms in the laboratory, and have shown metabolic recovery after decades of cell desiccation [14, 15]. However, the viability of naturally-desiccated microorganisms hundreds to thousands of years old in the McMurdo Ice Shelf [6] and McMurdo Dry Valleys [11] extends microbial dormancy up to thousands of years. Yet, the identity of the microorganisms surviving thousands of years of desiccation in a dormant state, and their molecular adaptations to overcome dryness and extremely low temperatures, are poorly understood.

The ability of microbes to thrive under extreme cold involves molecular adaptations, mostly aimed at synthesizing cryoprotective compounds (e.g., antifreeze proteins, compatible solutes or exopolysaccharides) [16] or changing the lipid composition of cell membranes to make them more fluid [17, 18]. Fatty acids are structural components of phospholipids, the primary building blocks of cell membranes in bacteria and eukaryotes [19]. Archaea rarely produces fatty acids [20], so their phospholipids incorporate isoprenoid chains [21, 22]. The main function of fatty acids in bacteria and eukaryotes is to provide membrane structure and regulate viscosity, which determines membrane permeability to molecules, active solute transport, as well as protein-protein interactions [18]. Regulation of membrane viscosity through changes in fatty acid composition (referred to as homeoviscous adaptation) allows organisms to maintain homeostasis and grow adequately in a changing environment [18]. Generally, a decrease in temperature causes a decrease in the average chain length of fatty acids, and an increase in double bonds (i.e., unsaturation), methyl branching and the ratio of *anteiso*- over *iso-* fatty acids [17, 23–25]. By contrast, an opposite trend with higher proportion of saturated versus unsaturated and/or branched fatty acids has also been described in bacteria exposed to extremely low temperatures [26, 27], making it difficult to generalize trends on bacteria and rather suggesting a species-specific response to temperature [28].

Beyond fatty acids, other lipid compounds such as alcohols and hydrocarbons are also part of bacterial lipid membranes and play a role in their stability [29, 30]. Sterols, although mainly components of eukaryotic cells involved in membrane fluidity and stress tolerance [31–33], have also been found sporadically in bacteria [33, 34], possibly by the acquisition of sterol biosynthetic pathways from eukaryotes through horizontal gene transfer [35]. In addition, squalenes are precursors of sterols and bacterial hopanoids [36, 37], which intercalate into lipid bilayers to alter the biophysical properties of membranes by decreasing their permeability [37]. While changes in bacterial fatty acids with temperature have been and continue to be studied in detail [17, 22, 24, 28], the influence of temperature on bacterial lipid composition beyond fatty acids, including alcohols and hydrocarbons, is less clear, yet they may provide clues about chemical adaptation to extreme cold. In addition, it remains unclear the taxonomic level (from phylum to species or even strain) at which bacterial lipid profiles remain stable under temperature variations.

Here we hypothesize that bacteria able to keep viability under extreme cold and dryness for hundreds of years exhibit remarkable lipid chemical richness, with fatty acids, alcohols and hydrocarbons all playing a role in bacterial survival and growth. In addition, we hypothesize that lipid profiles of cold-adapted or cold-tolerant bacteria vary at the strain level, as an intraspecific chemical diversity may allow greater survival at the population level under temperature changes. Therefore, the objectives of this study are (i) to isolate and identify viable bacteria from an ancient and desiccated microbial mat exposed to extreme cold and dryness for hundreds of years, (ii) to assess changes in the lipid (fatty acids, alcohols and hydrocarbons) composition and concentration in the isolated bacteria as a function of temperature, and (iii) to evaluate whether bacterial lipid profiles are similar or distinct between species and strains. The results of this study provide insights into the limits of bacterial survival in nature under extreme cold and aridity over hundreds of years, as well as identify bacterial lipid profiles to cope with low temperatures, which have biotechnological and astrobiological implications.

## Materials and Methods

### Sample Description and Bacterial Isolation

A naturally desiccated ancient microbial mat was collected in the McMurdo Ice Shelf (Antarctica) (78°00′S, 165°35′E) during the austral summer season of 1996 [6] (Fig. S1). The microbial mat was stored in dark and room temperature until the sterile plastic bag that contained it was opened under sterile conditions in 2015 for radiocarbon dating and physicochemical and mineralogical characterization [38]. The microbial mat was dated to ~1,000 years BP (Before Present) [38]. Then, in 2019, the sterile plastic bag with the microbial mat was opened again in sterile conditions for biological characterization using DNA metabarcoding, metaproteomics, lipid biomarker analysis, microscopy and microbial viability tests [6]. The viability of heterotrophic bacteria in the ancient mat was tested on solid R2A and LB media under different temperatures (15 ºC, 20 ºC and 25 ºC) [6]. Given the millennial age of the microbial mat, these viable bacteria have endured hundreds of years of thermal fluctuations. This prolonged exposure to temperature changes makes them relevant for assessing their lipid composition and thermal adaptations.

**Fig. 1:**
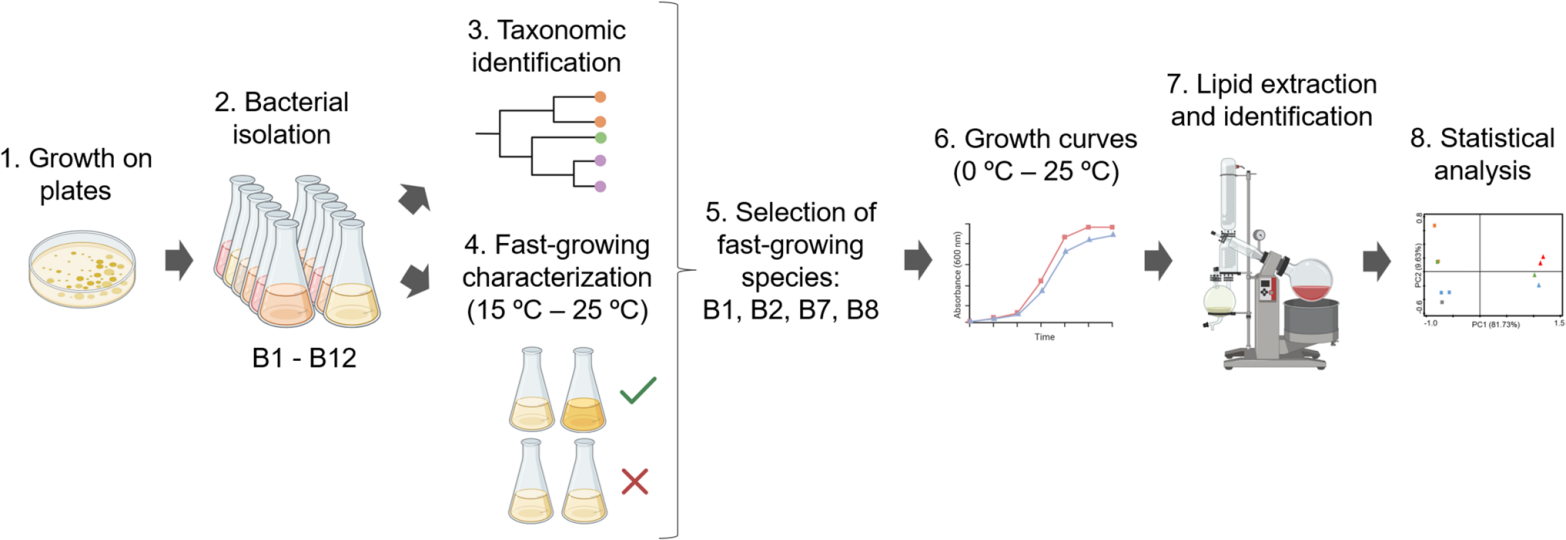
Methodological scheme to assess the influence of temperature on the lipid composition of extremophilic bacteria isolated from a 1,000-year-old microbial mat from Antarctica. 1. Bacterial growth on plates and colony selection; 2. Bacterial isolation of 12 strains (B1-B12, based on distinct colour and morphology) on liquid and solid R2A or LB media; 3. Taxonomic identification of the 12 bacterial strains by 16S rRNA gene analysis; 4. Identification of fast-growing bacteria by incubating the 12 isolates at 15 ºC, 20 ºC and 25 ºC for two months on liquid R2A or LB media; 5. Selection of fast-growing bacterial strains of different species (i.e., *Paenisporosarcina macmurdoensis* B1 and B2, and *Arthrobacter* sp. B7 and B8); 6. Growth curves of the selected strains at distinct incubation temperatures (from 0 ºC to 25 ºC); 7. Lipid extraction of the selected strains incubated at their maximum and minimum growth temperatures, and 8. Multivariate statistical analysis. Figure made using BioRender software

In this study we isolate, identify, and characterize 12 bacterial strains that grew on R2A and LB solid media at 20ºC based on distinct colour and morphology of the colonies (Fig. 1). For the bacterial isolation, we purified the bacterial colonies by repeatedly transferring and streaking on plates with R2A or LB media (depending on its original isolation medium). Purified bacterial strains were named as B1-B12. Strains B1-B6 were cultivated in LB medium and strains B7-B12 were cultivated in R2A medium, each on their original isolation media. All bacterial isolates were cryopreserved in 15% glycerol for long-term maintenance at −80 ºC.

### Genomic DNA Extraction

Genomic DNA from the 12 bacterial isolates were extracted using PowerBiofilm kit (QIAGEN, Hilden, Germany) following the manufacturer’s instructions of the kit. Previously, bacterial strains B1-B12 were grown in 100 mL flasks containing 40 mL of liquid LB or R2A medium (depending on its original medium) for 3 days at 20 ºC, with agitation (90 rpm) and in dark conditions. Then, bacterial cultures were transferred to 50 mL tubes, and centrifuged at 15,000 x g for 10 minutes to recover the pellets. A total of 400 µL of sterile 0.01 M phosphate buffer saline (PBS) were added to the pellets, tubes were vortexed and each bacterial pellet was transferred with a pipette to the bead tubes of the PowerBiofilm kit. Before starting the DNA extraction, the bead tubes containing the bacterial samples were centrifuged at 15,000 x g for 10 minutes to remove the PBS. A negative control without sample was also included during the DNA extraction. Genomic DNA was dissolved in sterile Milli-Q water and quantified with a Qubit dsDNA BR Assay kit (Invitrogen, Thermo Fisher Scientific, Waltham, MA, United States). DNA concentration of bacterial cultures ranged from 60 to 536 ng·µL^−1^. The negative control of the extraction showed DNA concentration below the quantification limit of the kit (10 pg·μl^−1^), suggesting no contamination during the extraction procedure. Genomic DNA of the samples was stored at −20°C for downstream analysis.

### PCR and Sequencing of the 16S rRNA Gene

Genomic DNA of the 12 bacterial isolates (B1-B12) was used for PCR amplification of the 16S rRNA gene. PCR was performed using two universal primer sets: 27F/907R and 533F/1492R_l [39, 40]. PCR was conducted in 25 µL containing 0.20 µM of each primer, 1x PCR buffer (Invitrogen Platinum Taq DNA polymerase, Thermo Fisher Scientific), 1.5 mM of MgCl_2_, 1.5 units of Taq and 1 µL of DNA (60 ng). The amplification of the 16S rRNA gene with the two primer sets was performed using the PCR conditions described in Lezcano et al. (2016) [41] with modifications to accommodate the PCR kit. Briefly, the initial activation step was performed at 94 ºC for 2 min, followed by 35 cycles of 94 ºC for 30 s, 55 ºC for 45 s, and 72 ºC for 1 min. Final extension step was conducted at 72 ºC for 10 min. PCR products were separated by electrophoresis in 1.5 % agarose gel at 110 V for 1 hour and 20 minutes. Then, bands (one of ~880 bp and other of ~977 bp) were visualized on a ChemiDoc MP imaging system (BioRad). PCR products were purified using QIAquick PCR Purification kit (QIAGEN) following manufacturer’s instructions and sequenced at the Centro de Astrobiología (Madrid, Spain). Sequencing of DNA samples was conducted following Sanger method using the BigDye Terminator v3.1 kit (Applied Biosystems, Thermo Fisher Scientific). Reactions were purified with columns made of Sephadex G50 (Thermo Fisher Scientific) and then DNA fragments were sequenced in a 3730xl DNA Analyzer with 48 capillaries (Applied Biosystems, Thermo Fisher Scientific).

### Phylogenetic Analysis

**Fig. 2:**
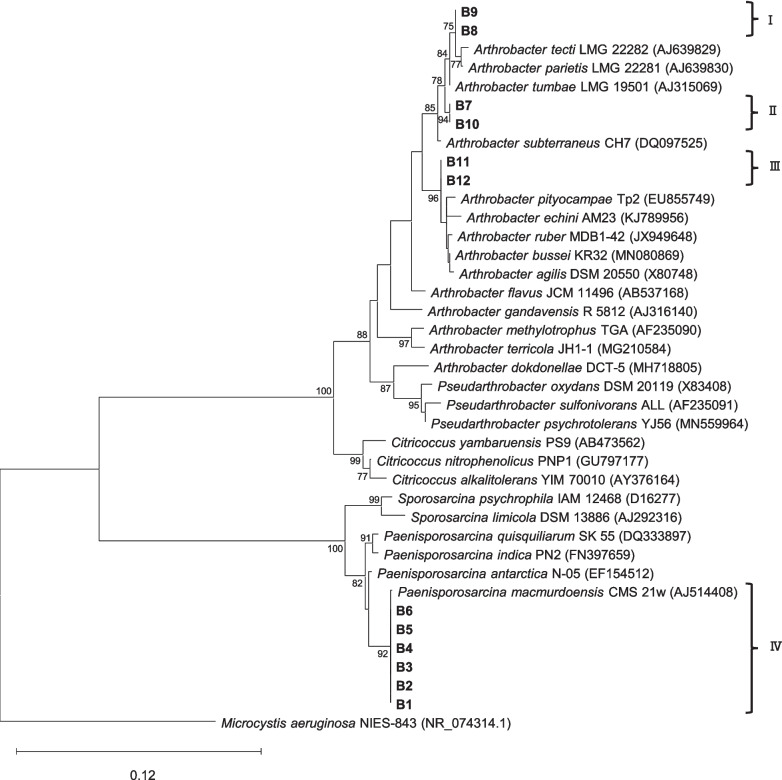
Taxonomic affiliations of the isolated bacterial strains (B1-B12). Maximum likelihood tree based on the 16S rRNA gene showing in bold the bacterial strains isolated in this study from the ancient microbial mat from the McMurdo Ice Shelf (Antarctica). Numbers near the nodes indicate bootstrap values greater than or equal to 70, as a percentage of 1000 replicates resulting from the phylogenetic analysis. The bar indicates 0.12 substitutions per nucleotide position. Clusters I, II and III include bacterial isolates B7-B12 and are identified with the genus *Arthrobacter*, while cluster IV comprises bacterial isolates B1-B6 and are identified with the species *Paenisporosarcina macmurdoensis*. GenBank accession numbers for the sequences used in the phylogenetic tree are provided in brackets

Final 16S rRNA gene sequences of the 12 bacterial isolates (B1-B12) were obtained from the alignment of four PCR sequences from two primer sets (including forward and reverse directions). Sequences were quality-filtered (end sequences were removed and errors were corrected) and combined (1103–1367 bp) using MEGA11 software [42]. DNA sequences were compared with those available in the EzBioCloud database [43] to establish a percentage of similarity. Additionally, a phylogenetic tree was constructed by performing a multiple sequence alignment with close related sequences using CLUSTAL W from MEGA11 software [42]. The phylogenetic tree was then generated using Maximum Likelihood method with the Tamura-Nei model and bootstrap analysis of 1,000 replicates in MEGA 11. The final tree showed the bacterial isolates distributed in different branches and clusters (I-IV) (“Bacterial taxonomic identification” from the Results section; Fig. 2). The 16S rRNA gene sequences of the 12 bacterial isolates (B1-B12) were deposited in the GenBank database under the following accession numbers: PQ814143-PQ814154.

### Bacterial Growth Assays at Distinct Temperatures

A subset of bacterial isolates from the original 12 was tested at 0 ºC, 5 ºC, 10 ºC, 20 ºC and 25 ºC to evaluate their ability to grow at different temperatures and identify their maximum and minimum temperatures for growth. The selection of the bacterial subset was based on distinct (i) taxonomy and position in the phylogenetic tree (Fig. 2), and (ii) growth rate in a pre-test performed in liquid R2A or LB media at 15 ºC, 20 ºC and 25 ºC for two months (Fig. 1). Strains B11 and B12 barely showed growth after two months of incubation (considered here as slow-growing bacteria) and thus were discarded for the growth assays. The remaining bacterial strains showed growth within two months of incubation (considered here as fast-growing bacteria), so two strains per taxon were selected: B1 and B2 (*Paenisporosarcina macmurdoensis*, from cluster IV), B7 (*Arthrobacter* sp., from cluster II), and B8 (*Arthrobacter* sp., from cluster I).

Prior to bacterial growth assays, bacterial strains were incubated in LB (strains B1 and B2) and R2A (strains B7 and B8) liquid media at 20 ºC, dark conditions, and agitation (90 rpm) until exponential phase (3-4 days). Then, bacterial inocula were transferred to liquid R2A or LB media in 100 mL flasks to obtain an initial absorbance of 0.01-0.03 measured at 600 nm in a spectrophotometer (BioSpectrometer fluorescence, Eppendorf, Hamburg, Germany). Growth assays of each bacterial strain were performed in triplicates at 0 ºC, 5 ºC, 10 ºC, 20 ºC and 25 ºC for 18 days in dark conditions and agitation (90 rpm). In addition, negative controls of liquid R2A and LB media without bacteria were included in triplicates. As bacterial strains B1 and B2 started to show growth beyond day 15 at 0ºC and 5ºC, growth assays for these strains were extended until day 21.

### Extraction and Analysis of Lipids

**Fig. 3:**
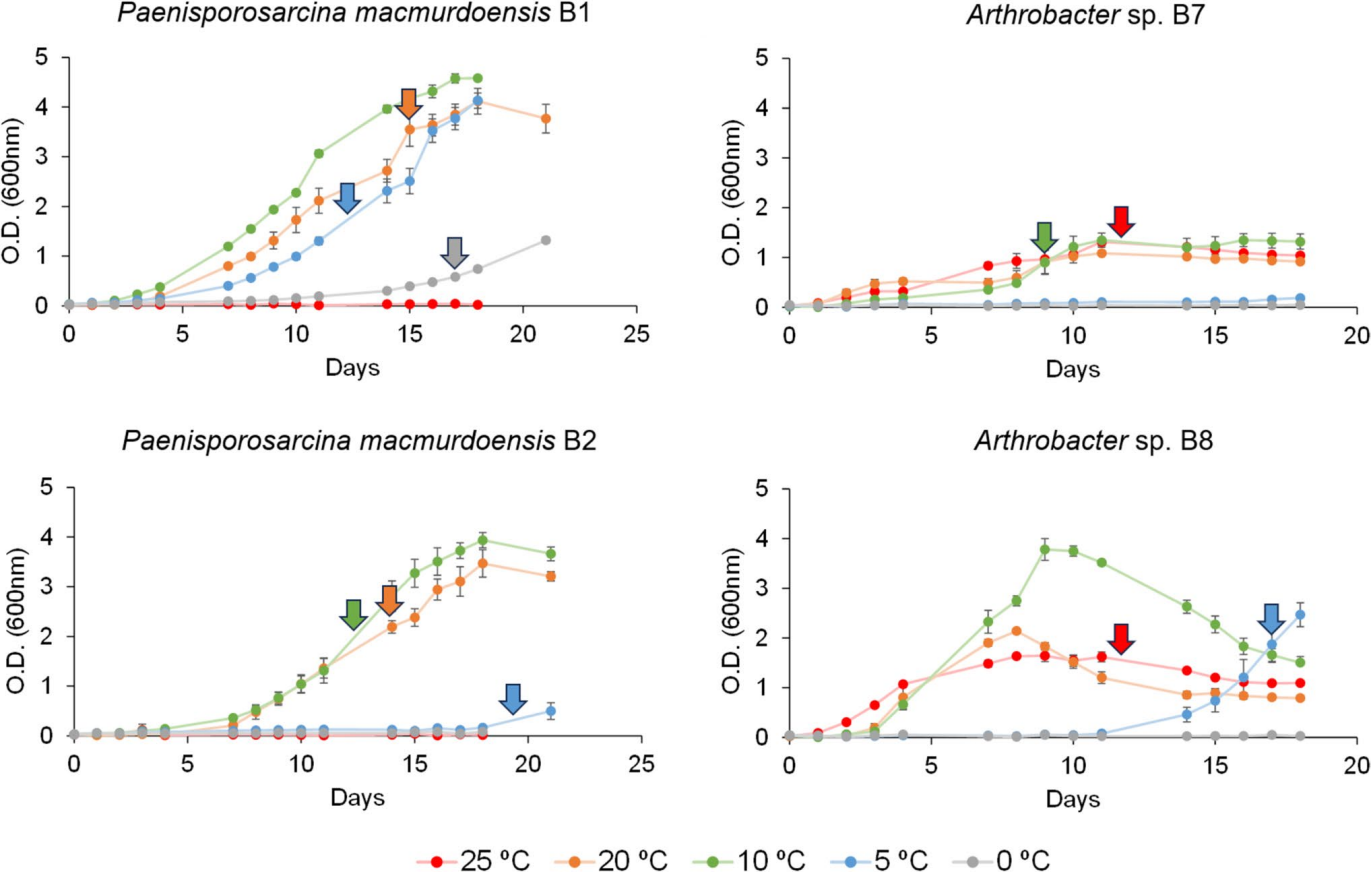
Growth of bacterial isolates as a function of incubation temperature. Growth curves of *Paenisporosarcina macmurdoensis* strains B1 and B2, and *Arthrobacter* sp. strains B7 and B8 under low (0 ºC, 5 ºC and 10 ºC) and warm (20 ºC and 25 ºC) temperatures up to 21 days of incubation under shaking and dark conditions. Coloured arrows indicate the optical density of each bacterial culture at the time of lipid extraction, corresponding to their early/mid exponential growth phase. Bars indicate standard deviation of triplicates. Media controls without bacteria showed no growth (Fig. S2)

We evaluated the effect of temperature on lipid composition and concentration of bacterial strains B1, B2, B7 and B8. Since bacteria thriving at temperatures that deviate from their optimal growth face the challenge of maintaining the functionality of their cells to stay alive [44], we incubated the strains B1, B2, B7 and B8 at their maximum and minimum survival temperatures (“Bacterial growth rates” from the Results section; Fig. 3). Therefore, strain B1 was incubated at 0 ºC and 20 ºC; strain B2, at 5 ºC and 20 ºC; strain B7, at 10 ºC and 25 ºC; and strain B8, at 5 ºC and 25 ºC. The growth rate of B1 at 0 °C and B2 at 5 °C were slow and, therefore, we also included an additional temperature of 5 ºC for B1 and 10 ºC for B2 to obtain a higher growth and ensure enough biomass for lipid analysis. The maximum survival temperatures (20 ºC and 25 ºC) serve as an extreme benchmark to compare lipid profiles at minimum survival temperatures (0ºC, 5ºC, and 10 ºC). While 0 ºC, 5 ºC, or 10 ºC are common on the McMurdo Ice Shelf, we cannot rule out the possibility of occasional temperature increases to 20 ºC or 25 ºC. This is supported by a recorded maximum temperature of 24.9 °C on the soil surface of the Fryxell basin in the McMurdo Dry Valleys [45] due to the incidence of solar radiation on the dark substrates (such as those on the McMurdo Ice Shelf, Fig. S1). Therefore, studying these temperatures provides a robust comparative framework for assessing adaptive changes in these Antarctic microorganisms.

Bacterial cultures were grown in 1 L flasks with 500 mL of LB (strains B1 and B2) or R2A (strains B7 and B8), in agitation (90 rpm) and dark conditions until they reached initial/mid exponential phase. Negative controls of LB and R2A medium without bacteria were also included. Once initial/mid exponential phase was reached, the bacterial cultures were centrifuged at 15.000 x g for 5 minutes to remove media and recover cell pellets. Pellets were then washed three times with 0.01 M PBS to remove traces of LB and R2A media. Bacterial pellets were freeze-dried and used as material for lipid extractions. A negative control was also included with 0.01 M PBS but without bacteria. Total biomass (mg dry weight) of bacterial pellets after incubation at the different temperatures are in Table S1. The total biomass of the negative control (containing freeze-dried PBS) was 9 mg.

Lipid extraction was performed using from 20 to 160 mg of freeze-dried pellet of each bacterium following a method previously described [46]. In addition, we also included the negative control (9 mg containing only PBS) to confirm the endogenous nature of the identified compounds in the bacterial strains. Briefly, samples were extracted using ultrasonication with a mixture of dichloromethane (DCM) and methanol (MeOH) at a volume proportion of 3:1, after addition of internal standards of hydrocarbons (*i.e.*, tetracosane-D_50_), fatty acids (*i.e*., myristic acid-D_27_), and alkanols (*i.e.*, 2-hexadecanol). The concentrated lipid extract (⁓1 mL) was hydrolyzed overnight with KOH (6% MeOH) at room temperature [47]. Then, a liquid-liquid extraction with *n*-hexane (3 cycles of 2 mL) was performed to obtain the neutral fraction. The remaining aqueous fraction was acidified with HCl (37%) to a pH value of 2. The acidic fraction containing the organic acids was recovered with *n*-hexane (3 cycles of 2 mL). Further separation of the neutral fraction into apolar (hydrocarbons) and polar (alcohols) fractions was conducted according to a method extensively described elsewhere [48].

All three polarity fractions were analyzed using gas chromatography coupled to mass spectrometry (GC-MS) from Agilent Technologies (8860 GC System and 5977B Mass Selective Detector), using a HP-5MS column (30 m x 0.25 mm i.d. x 0.25 μm film thickness) and He as a carrier gas at a constant flow of 1.1 mL·min^−1^. The analytical details of the GC oven are fully described elsewhere [46]. The apolar fraction was directly injected in the GC-MS tandem dissolved in *n*-hexane (1 μL), while the acidic and polar fractions needed to be previously derivatized respectively with methanolic BF_3_ and BSTFA to transform the fatty acids into methyl esters (FAME) and the alcohols into trimethyl silyl derivates [48]. The molecular identification of the three lipidic fractions was based on the use of reference materials (*i.e.*, isoprenoids, *n*-alkanes, *n*-fatty acids, mono- and poly-unsaturated alkanoic acids and *n*-alkanols) and the NIST library provided by the MSD ChemStation software (v. 01.03.2357, Agilent Technologies). For quantification, we used external calibration curves of *n*-alkanes (C_10_ to C_40_), FAMEs (C_8_ to C_24_) and *n*-alkanols (C_14_, C_18_, and C_20_). All compounds used as internal or external standards were supplied by Sigma Aldrich. Recovery of the internal standards was 77 ± 10 %.

### Statistical Analyses

Principal coordinates analysis (PCoA) based on the Bray Curtis dissimilarity distances was performed using the software CANOCO v.5.12 (Microcomputer Power, Ithaca, NY) to assess similarities in the bacterial lipid profiles at different temperatures. At this scope, we included in the analysis the concentration (µg · g^−1^ dw) of specific lipids from the apolar fraction (or hydrocarbons), acidic fraction (or fatty acids), and polar fraction (or alcohols) of each bacterial isolate (B1-B12) at their incubation temperatures. In addition, we performed a Permutational Multivariate Analysis of Variance (PERMANOVA) with 999 permutations based on the Bray-Curtis distance to assess whether differences in the lipid composition of strains are statistically influenced by temperature or bacterial taxonomy (i.e., *P. macmurdoensis* and *Arthrobacter* sp.). PERMANOVA analysis was performed using the “adonis2” function in the “vegan” package in R software [49].

## Results

### Bacterial Taxonomic Identification

A total of 12 pure bacterial strains, named as B1-B12, were isolated from a naturally desiccated ancient microbial mat collected in the McMurdo Ice Shelf (Antarctica). Colonies of white colour with transparent and smooth edges were named as strains B1-B6, those of yellow colour with smooth edges were named as B7-B10, and those of pink colour with smooth edges were named as B11 and B12. The analysis of the 16S rRNA gene and the construction of the phylogenetic tree with the closest 16S rRNA gene sequences in the EzBioCloud database showed four different clusters among the bacterial isolates (Fig. 2). Cluster I comprises strains B8 and B9; cluster II, strains B7 and B10; cluster III, strains B11 and B12; and cluster IV, strains B1, B2, B3, B4, B5 and B6. In each of these four clusters (I-IV), the 16S rRNA gene sequences of the bacterial isolates were identical (100 % similarity) to each other, suggesting that they were clonal in origin and thus are the same operational taxonomic unit (OTU).

Bacterial isolates B7, B8, B9 and B10 showed > 99 % similarity to *Arthrobacter tumbae* LMG 19501, *Athrobacter parietis* LMG 22281, *Athrobacter tecti* LMG 22282 and *Arthrobacter subterraneus* CH7 (similarity percentages of each strain are shown in Table S2), but they were located in two different clusters in the phylogenetic tree (Fig. 2). For this reason, strains B7, B8, B9 and B10 were classified up to the genus level as *Arthrobacter* sp. (phylum Actinomycetota, former Actinobacteriota). Bacterial strains B11 and B12 showed >99 % similarity to *Arthrobacter ruber* MDB1-42, *Arthrobacter agilis* DSM 20550, and *Arthrobacter bussei* KR32 (Table S2), but they were not located in the same cluster (Fig. 2). Therefore, bacterial strains B11 and B12 were also classified up to the genus level as *Arthrobacter* sp. By contrast, strains B1-B6 showed 99.9% similarity to *Paenisporosarcina macmurdoensis* CMS 21w, all grouping in the same cluster of the phylogenetic tree (Fig. 2). Therefore, strains B1-B6 were all classified as the species *Paenisporosarcina macmurdoensis* (phylum Bacillota, former Firmicutes).

### Bacterial Growth Rates at Different Temperatures

To evaluate the capacity of the bacterial isolates to grow at cold conditions and identify their maximum and minimum growth temperatures, we used a subset of 4 bacterial strains comprised of different species (“Bacterial growth assays at distinct temperatures”, from the Materials and methods section). We tested *P. macmurdoensis* B1 and B2, and *Arthrobacter* sp. B7 and B8, at 0 ºC, 5 ºC, 10 ºC, 20 ºC and 25 ºC (Fig. 3).

*P. macmurdoensis* B1 showed an optimal growth temperature at 10 ºC, and a minimum and maximum growth temperatures (i.e., the lowest and highest temperature at which the microorganism can grow) at 0ºC and 20 ºC, respectively (Fig. 3). *P. macmurdoensis* B2 showed the same optimum (10 ºC) and maximum (20 ºC) growth temperatures as strain B1, but the minimum was 5 ºC instead of 0 ºC. The bacterial strain *Arthrobacter* sp. B7 showed optimal growth at temperatures ranging from 10 ºC to 25 ºC, but was unable to grow below 10 ºC. *Arthrobacter* sp. B8 showed optimal growth temperature at 10 ºC, and a minimum and maximum growth temperatures at 5 ºC and 25 ºC, respectively. The minimum temperatures for bacterial growth are limited by the incubation period of our experimental design. Therefore, these results do not exclude the possibility that a bacterial strain can grow at low temperatures (e.g., strain B2 at 0 ºC) with a longer incubation time.

### Bacterial Lipid Composition and Concentration at Different Temperatures

To evaluate temperature-induced changes in the lipid composition and concentration of the isolated bacteria, we incubated *P. macmurdoensis* B1 and B2, and *Arthrobacter* sp. B7 and B8 at their maximum and minimum temperature for growth (Fig. 3). Therefore, we incubated *P. macmurdoensis* B1 at 0 ºC and 20 ºC, *P. macmurdoensis* B2 at 5 ºC and 20 ºC, *Arthrobacter* sp. B7 at 10 ºC and 25 ºC, and *Arthrobacter* sp. B8 at 5 ºC and 25 ºC. In addition, *P. macmurdoensis* strains B1 and B2 were incubated at 5 ºC and 10 ºC, respectively, to obtain a higher amount of biomass for lipid extraction (Table S1).

**Fig. 4:**
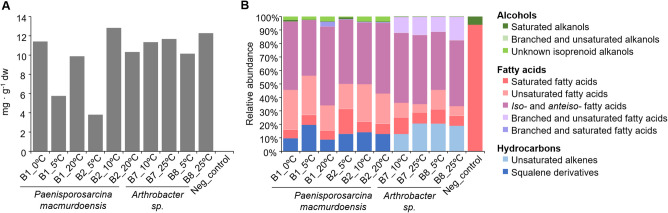
Lipid composition of bacterial isolates as a function of incubation temperature. **A**) Lipid concentration relative to dry weight (mg · g^−1^ dry weight) and **B**) composition and relative abundance of each type of lipid within hydrocarbons, fatty acids and alcohol families in the *Paenisporosarcina macmurdoensis* strains B1 and B2 and *Arthrobacter* sp. strains B7 and B8 after incubation at their maximum and minimum temperatures for growth. For strains B1 and B2, an additional temperature for each strain (5 ºC for B1 and 10 ºC for B2) was included to assure enough biomass for lipid extraction

In general, all bacterial strains showed similar total lipid content per cell dry weight at all temperatures, ranging from 10 to 13 mg · g^−1^ dw (Fig. 4A). Only *P. macmurdoensis* strains B1 and B2 grown at 5 ºC produced lower lipid concentrations (below 6 mg · g^−1^ dw) compared to the other temperatures. The negative control showed 0.01 mg · g^−1^ dw of total lipids, so that external contamination in the experiments was considered negligible.

**Fig. 5:**
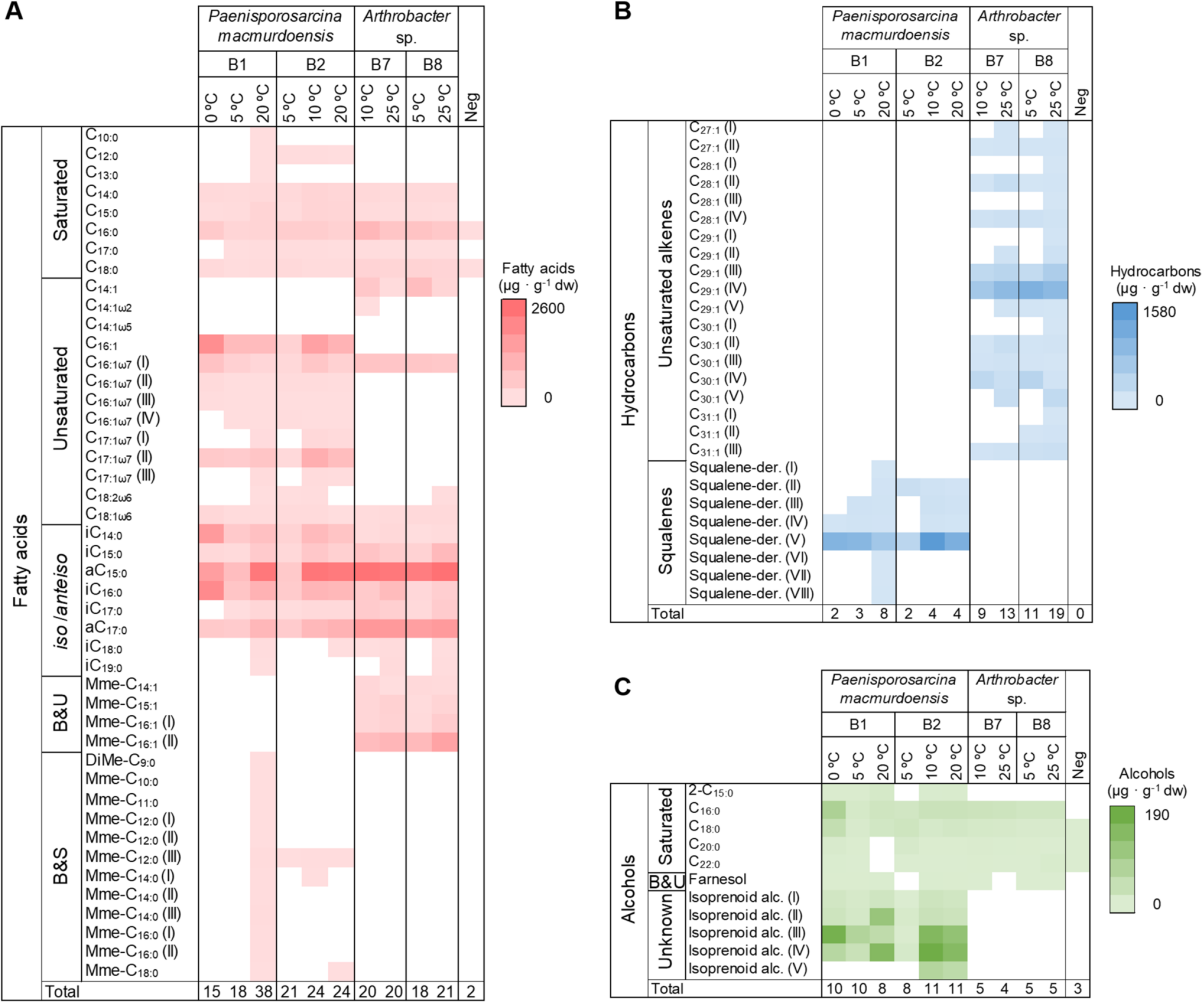
Specific lipid composition and concentration of bacterial isolates as a function of incubation temperature. Composition and concentration (µg · g^−1^ dry weight) of specific lipid compounds in *Paenisporosarcina macmurdoensis* strains B1 and B2 and *Arthrobacter* sp. strains B7 and B8 after incubation at their maximum and minimum temperatures for growth (Fig. 3). For strains B1 and B2, an additional temperature for each strain (5 ºC for B1 and 10 ºC for B2) was included to assure enough biomass for lipid detection. The negative control of the experiment (phosphate buffered saline medium without bacteria) is also included (i.e., “Neg”). In **A**), lipid compounds from the acidic fraction or fatty acids; in **B**), from the apolar fraction or hydrocarbons, and in **C**), from the polar fraction or alcohols. “B&U” means branched and unsaturated fatty acids or alkanols, “B&S” stands for branched and saturated fatty acids, and “DiMe” or “Mme” indicate dimethylated or monomethylated monounsaturated fatty acids. The position of the double bond in the “C_14:1_” and “C_16:1_” fatty acids is unknown. Roman numbers in brackets refer to different isomers of the same compound

Across all bacterial strains and temperatures, fatty acids represented the predominant lipid class (83 ± 4 %), followed by hydrocarbons (15 ± 5 %) and alcohols (2 ± 2 %) (Fig. 4B). The most abundant lipids in all strains are *iso* and *anteiso* (49 ± 5 %), unsaturated (18 ± 9 %) and saturated (9 ± 4 %) fatty acids. In particular, the most abundant and widely distributed fatty acids among the four strains at all temperatures were *i*C_14:0_, *i*/*a*C_15:0_, *i*C_16:0_, and *i*/*a*C_17:0_, with concentrations ranging from 11 to 2,602 µg · g^−1^ dw, and the straight chain or *normal* (*n*-) fatty acids from C_14:0_ to C_18:0_, with concentrations ranging from 5 to 922 µg · g^−1^ dw (Fig. 5A). In addition, a number of even *n*-alkanols from C_16_ to C_22_ (up to 81 µg · g^−1^ dw), and the isoprenoid farnesol (up to 2.7 µg · g^−1^ dw), were also identified in most bacterial strains at all temperatures (Fig. 5C).

Despite the general similarities in the acidic fraction between the four strains at all temperatures tested, *P. macmurdoensis* and *Arthrobacter* sp. strains showed clear differences in the composition and relative abundance of specific fatty acids, hydrocarbons and alcohols (Fig. 5), supported by PERMANOVA analysis (*p* < 0.05, R^2^= 0.5259) (Table S3). *P. macmurdoensis* strains B1 and B2 showed relatively high concentration of unsaturated fatty acids of 16, 17 and 18 carbons, such as C_16:1(ω7)_, C_17:1(ω7)_, C_18:1ω6_, and C_18:2ω6_, while *Arthrobacter* sp. strains B7 and B8 contained only trace amounts of the acids C_16:1ω7_ and C_18:1ω6_ (Fig. 5A, Table S4). In addition, squalene derivatives (13 ± 4 %) (Fig. 5B, Table S5) and isoprenoid alcohols (3 ± 1 %) (Fig. 5C, Table S6) were only detected in *P. macmurdoensis* strains B1 and B2, while were not detected in the *Arthrobacter* sp. strains B7 and B8. By contrast, *Arthrobacter* sp. strains B7 and B8 showed a wide variety of unsaturated alkenes (1-alkenes) from C_27:1_ to C_31:1_ (18 ± 4 %) that were not detected in the *P. macmurdoensis* strains (Fig. 5B, Table S5). Similarly, monomethylated-monounsaturated fatty acids of 14 to 16 carbons (Mme-C_14:1_ to Mme-C_16:1_) were only identified in the *Arthrobacter* sp. strains (Fig. 5A, Table S4).

**Fig. 6:**
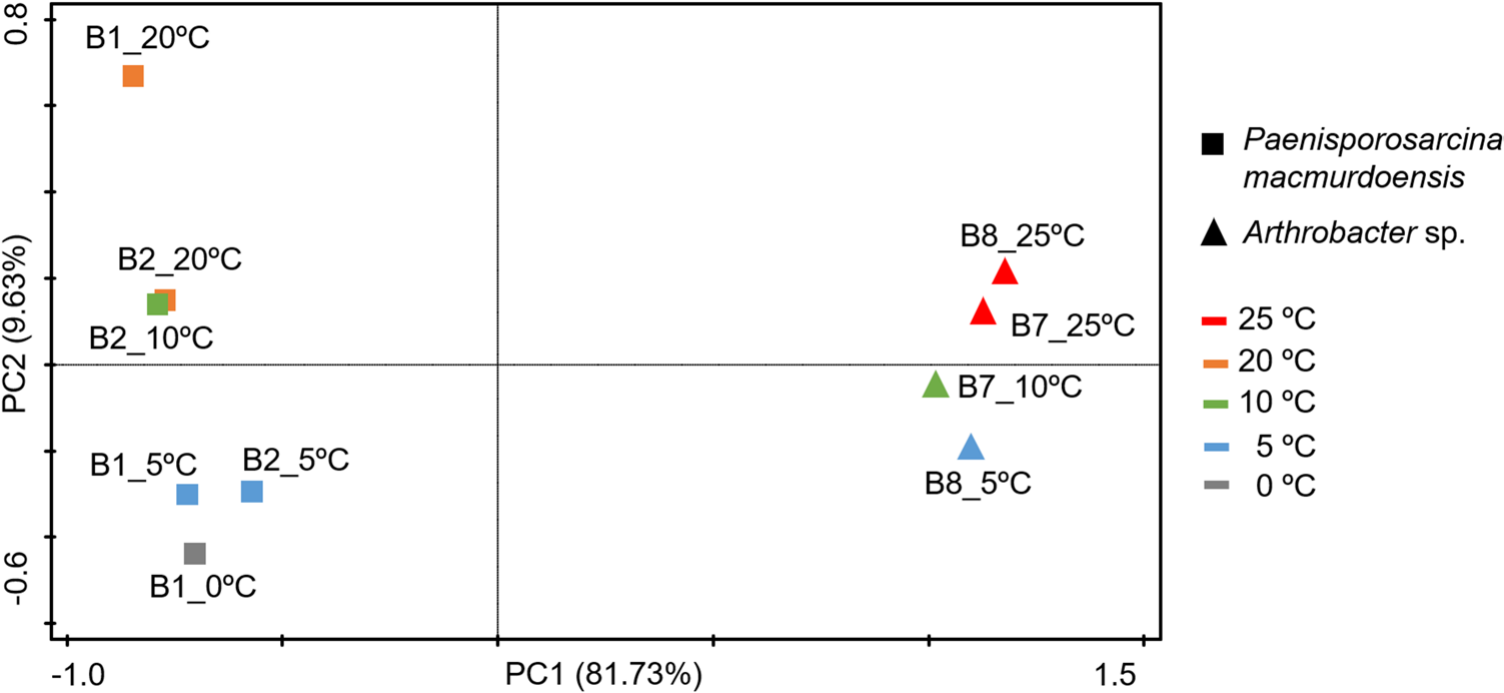
Similarities/dissimilarities in the lipid profile of bacterial isolates as a function of taxonomy and incubation temperature. Principal Coordinates Analysis (PCoA) based on the Bray-Curtis dissimilarity distances using the concentrations (µg · g^−1^ dw) of the specific lipid compounds produced by *Paenisporosarcina macmurdoensis* strains B1 and B2, and *Arthrobacter* sp. strains B7 and B8 incubated at their maximum and minimum temperatures for growth (Fig. 5)

The composition and abundance of membrane lipids was mainly driven by taxonomy, as evidenced by the PCoA plot, which distinctly separates the lipid profiles of *P. macmurdoensis* and *Arthrobacter* sp. strains in two groups irrespective of incubation temperature (Fig. 6). Although lipid profiles were primarily associated with taxonomy, temperature also influenced lipid composition in *P. macmurdoensis* and *Arthrobacter* sp. This is evidenced by the separation of lipid profiles by temperature incubation in both taxa along the second principal coordinate in the PCoA plot (Fig. 6). These results are supported by the PERMANOVA analysis (Table S3), which revealed a statistically significant influence of both temperature (*p*=0.039, R^2^= 0.1759) and, more strongly, taxonomy (*p*=0.004, R^2^=0.5259) on lipid profiles. However, the scarcity of measurements for temperature treatment and, therefore, the inability to adequately assess intra-treatment variability, lead us to interpret these results with caution.

The lipid response of *P. macmurdoensis* strains B1 and B2 to temperature changes was different even though they belong to the same species. The lipid profiles of strain B1 showed higher richness of fatty acids, hydrocarbons and alcohols at 20 ºC as compared to those at 0 ºC and 5 ºC (i.e., 18 fatty acid compounds at 5 ºC versus 38 at 20 ºC) (Fig. 5). By contrast, the lipid richness at low and high temperatures in strain B2 was similar (e.g., 21 fatty acids compounds at 5 ºC versus 24 at 10 ºC or 20 ºC) (Fig. 5A). Among these two strains, the major lipid changes as a function of incubation temperature were unsaturated, *iso-* and *anteiso-*, and other branched fatty acids (Fig. 5A, Table S4), squalene derivatives (Fig. 5B, Table S5) and some unidentified alcohols (Fig. 5C, Table S6). For instance, strain B1 grown at 0 ºC yielded a relatively higher concentration of monounsaturated fatty acids of 16 carbons, *i*C_14:0_, *i*C_16:0_ and some unidentified alcohols, while the strains B1 and B2 grown at 20 °C showed a relatively higher proportion of *a*C_15:0_, *a*C_17:0_, *i*C_18:0_, *i*C_19:0_, mono- and di-methylated-monounsaturated fatty acids, and some unidentified alcohols.

## Discussion

### Bacteria Surviving Freezing and Desiccation in a ~1,000-Year-Old Microbial Mat

Scarcity of water is a challenge for the survival of microorganisms in hot and cold deserts and can be incompatible with life. The minimum water availability for biological functions is described as a water activity (a_w_) limit and has been shown to be organism-specific [14, 50]. While most microorganisms require a_w_ >0.9 [50], extremophilic microorganisms can grow at lower a_w_ values, such as 0.635 in Haloarchaea or 0.640 in the fungus *Xeromyces bisporus* [50]. The completely freeze-dried microbial mat of the McMurdo Ice Shelf is expected to have 0 or near 0 g H_2_O g^−1^ of dry weight, preventing the circulation of solutes and thus any microbial metabolism. In these conditions, long-term survival of microorganisms in the microbial mat depends on the presence of resistant cell types and/or specific molecular adaptations that enable persistence during freezing and desiccation. Although we cannot completely rule out the possibility of minor rewetting events since the microbial mat dried, the old age of the mat (~1,000 years BP), the location where it was found (on the slope of a hill), and the climatic conditions of the area (dry and cold), suggest that biological activity has been minimal or absent for much of that time [6]. Yet, the desiccated microbial mat showed viable bacteria when rewetted and incubated on plates with R2A (26 ± 6 CFU mg^−1^ dw) and LB (1.0 ± 0.9 CFU mg^−1^ dw) media at 20 ºC [6]. Among these colonies, in the present study we isolated 12 pure bacterial strains named B1-B12. The isolation of these 12 bacterial strains demonstrates their outstanding tolerance to freezing and desiccation for hundreds of years and suggests the presence of molecular adaptations that have allowed metabolic reactivation once rewetted.

Taxonomic analysis of the 16S rRNA gene of the bacterial strains B1-B6 showed 99.9 % sequence similarity to the 16S rRNA gene of *Paenisporosarcina macmurdoensis* CMS 21w, isolated from a cyanobacterial mat of a pond in the Wright Valley, located in the McMurdo Dry Valley region (Antarctica) [51]. The McMurdo Dry Valley is the largest area of ice-free ground in the Antarctic continent [7], harbouring ephemeral ponds and streams under extremely low temperatures (e.g., mean annual air temperature of −17 ºC) and scarce precipitation (<100 mm water equivalent) [52]. Although the McMurdo Dry Valley differs from the McMurdo Ice Shelf in the type of surface (land extension versus a large platform of floating glacial ice, respectively) [6, 53], their proximity (~100-150 km in a straight line), the shared presence of seasonal ponds and the similar environmental conditions (i.e., extremely low temperatures and precipitation) may explain the distribution of *P. macmurdoensis* in both places. Indeed, the optimum growth of *P. macmurdoensis* strains B1 and B2 was observed at 10 ºC, with capacity to thrive at 0 ºC and inability to grow above 20 ºC (Fig. 3), which classify the strains as psychrophilic (i.e., cold-adapted bacteria) [1] and circumscribes their distribution to extremely cold environments. For instance, the genus *Paenisporosarcina* is commonly distributed in water and soils from Antarctica [51, 54], and it has also been found in agricultural soils in humic continental climates [55].

In addition to cold adaptation, the capacity of spore production in *P. macmurdoensis* [51] possibly explains the tolerance of this species to desiccation over hundreds of years. Furthermore, the presence of xeroprotectants, such as trehalose or lipase, are known to contribute to desiccation tolerance in other bacteria [56]. Future research involving the analysis of possible xeroprotectants in *P. macmurdoensis* could provide new clues about its tolerance to desiccation.

Unlike *P. macmurdoensis* strains, *Arthrobacter* sp. strains B7 and B8 showed wider temperature tolerance. Optimal growth of *Arthrobacter* sp. strain B7 ranged from 10 ºC to 25 ºC, and that of strain B8 was at 10 ºC with the ability to thrive from 5ºC to 25ºC (Fig. 3) Therefore, both strains classify rather as psychrotrophic (i.e., cold-tolerant bacteria) [1] with a broad temperature tolerance that may confer them a global ecological distribution. For instance, the genus *Arthrobacter* has been found in a huge variety of environments, including soils, fresh and sea water, air, volcanic rocks, mural paintings, cryoconites, guano, and even human blood and skin, among others [57]. Particularly, *Arthrobacter* sp. strains B7 and B8 showed >99 % sequence similarity to the 16S rRNA gene of *Athrobacter tumbae* LMG 19501, *Athrobacter parietis* LMG 22281, and *Athrobacter tecti* LMG 22282 (Table S2), all isolated from mural paintings of the Servilia tomb in the Roman necropolis of Carmona (Spain) and Saint-Catherine chapel (castle at Herberstein, Austria) [58]. The environmental conditions at these sites are very different from those at the McMurdo Ice Shelf (i.e., higher temperatures and precipitation). In addition, all *Arthrobacter* sp. strains isolated in this study and that of Heyrman et al. (2005) [58] shared the common characteristic of originating from ancient samples that have endured hundreds of years of variable environmental conditions, suggesting a high metabolic versatility of *Arthrobacter* sp. B7 and B8.

By contrast, *Arthrobacter* sp. strains B11 and B12 showed 99.77 % similarity to *Arthrobacter ruber* MDB1-42 (Table S2), a bacterium isolated from ice in the Midui glacier in Tibet [59], and barely showed growth in liquid medium for at least two months at 15 ºC, 20 ºC or 25 ºC. The low capacity of *Arthrobacter* sp. strains B11 and B12 to grow in liquid media (as opposed in solid media), makes them much less versatile than *Arthrobacter* sp. strains B7 and B8, suggesting a preference for a surface-attached rather than a free-living lifestyle. Overall, the viability and tolerance of *Arthrobacter* sp. B7, B8, B11 and B12 to hundreds of years of desiccation may be explained by its rod-coccus growth cycle, in which the coccus phase offers stability and allows tolerance to environmental changes [60].

The isolation of *Arthrobacter* (phylum Actinomycetota) and *Paenisporosarcina* (phylum Bacillota) strains aligns with the detection of both genera in the ancient microbial mat through massive 16S rRNA gene sequencing [6]. However, these genera were found in low relative abundance in the microbial mat (0.11 % for *Arthrobacter* and 0.07 % for *Paenisporosarcina*; Table S7). By contrast, other genera such as *Nocardioides* (3.78%, Actinomycetota) and *Desulfosporosinus* (26.07%, Bacillota), were highly abundant in the desiccated mat (Table S8) but were not isolated in the laboratory under our experimental conditions. This discrepancy between the dominant bacterial genera in the mat and the difficulty in their isolation may be due to some factors: (i) DNA-based analyses may not accurately reflect the viable community, which is particularly relevant in this ancient and desiccated microbial mat, and (ii) cultivating microorganisms in the laboratory is challenging, especially those from extreme environments where replicating complex natural conditions is difficult [61].

### Relationship of Bacterial Lipid Profiles to Taxonomy and Temperature

In the psychrophilic *P. macmurdoensis* strains B1 and B2, changes in total lipid content were observed as a function of the incubation temperature (Fig. 4A). In these strains, the total concentration of lipids was lower at 5 ºC (5 ± 1 mg · g^−1^ dw) than at 0 ºC, 10 ºC and 20 ºC (mean ± sd: 11 ± 1 mg · g^−1^ dw). Indeed, the fact that the low lipid concentrations at 5 °C were not maintained at 0 ºC suggests differential responses of strains B1 and B2 to low temperatures, rather than a stable adaptation to cold. These results differ from those of Akulava et al. (2024) [28], who explored the lipid content and composition of 74 psychrophilic bacterial strains of different genera and observed a remarkable stability in their lipid concentration between 5 ºC and 25 ºC. Therefore, the lowest lipid concentration at 5 ºC in strains B1 and B2 suggests this temperature as a tipping point in this species, where lipid production may decrease in favour of other compounds to cope with freezing. Production of cryoprotective compounds (e.g., antifreeze proteins or exopolysaccharides) in psychrophilic bacteria has already been described [16, 62], and is usually highest at temperatures close to 0ºC or below [63]. However, the lack of analysis of exopolysaccharides or antifreeze proteins in this study prevents testing this hypothesis.

Most studies on bacterial lipids have focused on fatty acid composition and abundance [24, 26, 28, 64] and have used fatty acid profiles to ascribe taxonomy and even Gram type [28]. Here we present more comprehensive lipid profiles which include not only fatty acids, but also alcohols and hydrocarbons, providing more detailed information that helps infer taxa more accurately. For instance, the bacterial genera *Paenisporosarcina* and *Arthrobacter* have similar proportions of saturated (0.4 - 1.4 % dw), unsaturated (0.7 - 3.6 % dw), and *iso-* and *anteiso-* (1.8–6.0 %) fatty acids, but differ in their composition and abundance of other lipids, such as unsaturated alkenes in *Arthrobacter* (up to 21% dw), or squalene derivatives (up to 20 % dw) and unidentified isoprenoid alcohols (up to 4% dw) in *Paenisporosarcina* (Fig. 4). Therefore, despite both genera being Gram-positive [51, 57] and having a similar composition and abundance of major fatty acids groups, their lipid profiles are clearly different when considering hydrocarbons and alcohols. These differences in the composition and abundance of lipid profiles appear to be taxon-specific and, accordingly, *Arthrobacter* and *Paenisporosarcina* strains clearly grouped separately in the PCoA plot, regardless of the incubation temperature (Fig. 6). This finding is supported by the PERMANOVA analysis (*p* < 0.05, R^2^= 0.5259), which indicates that 52.59% of the variance in lipid profiles can be explained by taxonomy. Therefore, the differences in lipid profiles between the two genera suggest that taxonomy may be a more influential factor than temperature in shaping the composition and abundance of membrane lipids. It should be noted that the use of two distinct culture media could partially influence the lipid profile [65, 66]. However, the coincidence of predominant fatty acids in our *Arthrobacter* (e.g., *a*C_15:0_, *a*C_17:0_ and *i*C_16:0_) and *Paenisporosarcina* (e.g., *a*C_15:0_ or *a*C_17:0_) strains with those of other *Arthrobacter* and *Paenisporsarcina* strains suggests a relatively low influence of the culture medium [24, 67].

Despite the taxon-associated lipid composition, the incubation temperature also influenced the lipid profiles in *P. macmurdoensis* and *Arthrobacter* sp. strains, a finding supported by the PERMANOVA analysis (*p* < 0.05, R^2^= 0.1759), which indicates that 17.59% of the variance can be attributed to temperature. The scarcity of observations per temperature treatment means that this interpretation has to be considered with caution. Lipid variability with temperature was especially remarkable in *P. macmurdoensis*, where strains B1 and B2 showed a separation of their lipid profiles in the PCoA plot when incubated at 0 ºC and 5 ºC from those at 10 ºC and 20 ºC (Fig. 6). Similarly, *Arthrobacter* strains B7 and B8 also showed differences in their lipid profiles when incubated at 25 °C from those at 10 °C or 5 °C (Fig. 6). The general lower proportion of *anteiso-* over *iso-* fatty acids and the absence of mono- and di-methylated monounsaturated fatty acids in *P. macmurdoensis* strains B1 and B2 at the lowest temperature (Fig. 5A) suggest that these fatty acid compounds may not play an essential role in increasing membrane viscosity at cold temperatures in this species. This is in contrast with the general observations of increasing fatty acid unsaturation, methyl branching and the ratio of *anteiso-* over *iso-* fatty acids with temperature decrease [17, 23, 24].

Beyond fatty acids, alcohols and hydrocarbons also showed qualitative and quantitative differences with temperature in *P. macmurdoensis*. A higher proportion of isoprenoid alcohols and a higher diversity of squalene derivatives in the two strains at temperatures above 10 ºC than at 0 ºC or 5ºC (Fig. 5) suggest a role for these compounds in decreasing membrane permeability with increasing temperature. For instance, squalene derivatives are precursors of sterols and bacterial hopanoids [36, 37] that intercalate into lipid bilayers to condense, thicken and decrease the permeability of membranes [37]. In addition, unsaturated alkenes from 27 to 31 carbons appear to contribute to the permeability of the membrane of *Arthrobacter* strains at high temperatures, where the richness of these compounds was higher at 25ºC than at 5ºC or 10ºC (Fig. 5B). Although reports on long-chain polyunsaturated alkanes in bacteria are scarce, they have been described as common membrane components of *Chloroflexus aurantiacus* and other thermophilic and mesophilic relatives from the Chloroflexota phylum [68]. In addition, the long-chain polyunsaturated hydrocarbon hentriacontanonene (C_31:9_) was identified in a psychrophilic bacterium isolated from Antarctic sea ice cores in Prydz Bay [69], and also in a marine *Shewanella* bacterium (phylum Pseudomonadota) able to grow at 4 ºC [70]. Although the specific role of long-chain unsaturated alkanes in our *Arthrobacter* strains is unclear, their greater concentration at the highest temperature may be related to the regulation of membrane fluidity to increasing temperatures to ensure proper membrane function under thermal stress [44]. Therefore, our results suggest that the influence of temperature on the lipid profile is not homogeneous in bacteria, and it is in agreement with other studies that have shown different lipid composition among bacterial genera exposed to low temperatures [26–28, 44].

### Intraspecific Lipid Diversity may Favour Bacterial Adaptation to Temperature Fluctuations

Besides the clearly distinct lipid profiles between the two bacterial genera studied here, the incubation temperature also influenced the lipid composition and abundance at the strain level. This intraspecific variation of lipid profiles with temperature was observed in *P. macmurdoensis*, where strains B1 and B2 showed a clear separation of their lipid profiles when incubated at 20 ºC (Fig. 6). For instance, *P. macmurdoensis* strain B1 shows higher fatty acid richness −of mostly methylated monounsaturated fatty acids (DiMe-C_9:0_, Mme-C_10:0_, Mme-C_11:0_, Mme-C_12:0_, Mme-C_14:0_, Mme-C_16:0_ and Mme-C_18:0_)− than strain B2 at 20 °C (Fig. 5A). We hypothesize that this intraspecific lipid variability may be an adaptive response to favour survival at the population level, as a consortium of strains of the same species with different lipid composition may favour population survival under drastic and rapid environmental changes, such as a rise or decrease in temperature. A diversity of fatty acids and other lipids in cell membranes has also been observed in different strains of *Corynebacterium* species, and has been explained as an adaptive advantage for inhabiting different ecological niches [71]. Therefore, intraspecific lipid diversity in *P. macmurdoensis* likely facilitated adaptation to fluctuating temperatures, contributing to its survival in the ancient microbial mat over 1,000 years.

Despite the ecological benefits of bacterial lipid heterogeneity at the strain level, the intraspecific lipid diversity and the instability of lipid profiles as a function of temperature can easily lead to taxonomic misidentification in environmental samples. Although lipids are not used as the main criterion for identifying bacteria at the genus or species level because many lipids are widely distributed in different taxonomic groups, they are among the most recalcitrant molecular markers key for identifying life remnants in old environmental samples [72–75]. Unlike DNA, which has high taxonomic resolution but relatively low preservation rates in the environment (up to 1 million year [76]), lipids have lower taxonomic resolution but withstand millions and billions of years in the geological record [74]. Consequently, lipid biomarkers are widely used in paleobiology [6, 77] and astrobiology [78] for taxonomic identification. Therefore, characterizing the lipids of isolated bacteria under key environmental variables, such as temperature, expands taxon-associated lipid inventories and enhances paleobiological interpretations in ancient samples from Earth and beyond.

## Conclusion

This study demonstrates the viability of *Paenisporosarcina macmurdoensis* and *Arthrobacter* sp. from a naturally desiccated 1,000-year-old microbial mat from Antarctica. We isolated and cultured 12 bacterial strains, comprising 6 of each taxon as determined by 16S rRNA gene analysis. Then, we exposed 2 strains of each taxon to their minimum and maximum growth temperatures to evaluate changes in their lipid profiles. The two genera exhibited taxon-specific lipid profiles, but incubation temperature also influenced the lipid composition of fatty acids, hydrocarbons and alcohols. *P. macmurdoensis* strains showed a distinct lipid composition at 0 °C and 5 °C compared to that at 20 °C, with the concentration of methylated monounsaturated fatty acids (e.g., DiMe-C_9:0_, Mme-C_12:0_ or Mme-C_14:0_) increasing with temperature. In addition, the two *P. macmurdoensis* strains had distinct lipid composition when incubated at 20 ºC. This intraspecific lipid diversity likely provides an adaptive advantage to thermal fluctuations in the mat, thus favouring species survival. While strain-level lipid diversity may offer ecological advantages for certain species survival (i.e., *P. macmurdoensis*), this intraspecific lipid heterogeneity may cause microbial misidentification in environmental ancient samples, where lipid biomarkers are crucial for detecting traces of past life. This highlights the need for a comprehensive assessment of species-specific lipid diversity under key environmental variables, such as temperature, to expand taxon-associated lipid inventories and improve biological interpretations of ancient terrestrial and extraterrestrial samples.

## Supplementary Information

Below is the link to the electronic supplementary material.

Supplementary file1 (DOCX 822 KB)

## Data Availability

DNA sequence data is available at the NCBI GenBank database under the accession numbers PQ814143-PQ814154.
