# Supplementary material for: Temperature-Sensitive Lipids Reveal Intraspecific Diversity in Bacteria Isolated from an Ancient Antarctic Microbial Mat
Source: Microb Ecol. 2025 Jul 31;88:84. doi: 10.1007/s00248-025-02583-4 (PMC12313772; doi:10.1007/s00248-025-02583-4)
Supplement: Supplementary file 1 [file 248_2025_2583_MOESM1_ESM.docx]

**Supplementary material**

**Temperature-sensitive lipids reveal intraspecific diversity in bacteria isolated from an ancient Antarctic microbial mat**

María Ángeles Lezcano^1,2*^, Daniel carrizo^1^, Miguel Ángel Lominchar^1^, Laura Sánchez-García^1*^, Antonio Quesada^1,3^, Víctor Parro^1^

^1^ Centro de Astrobiología (CAB), CSIC-INTA, 28850 Torrejón de Ardoz, Madrid, Spain

^2^ IMDEA Water Institute, Avenida Punto Com 2, 28805 Alcalá de Henares, Madrid, Spain

^3^ Departamento de Biología, C. Darwin 2, Universidad Autónoma de Madrid, Madrid, Spain

*Corresponding authors: María Ángeles Lezcano ([mangeles.lezcano@gmail.com](mailto:mangeles.lezcano@gmail.com), [mangeles.lezcano@imdea.org](mailto:mangeles.lezcano@imdea.org)), Laura Sánchez-García (lsanchez@cab.inta-csic.es)

**Fig. S1** A) View of Bratina Island (McMurdo Ice Shelf, Antarctica) showing the abundant meltwater ponds. B) A 1,000-year-old desiccated microbial mat collected from the shore of one of the ponds on Bratina Island [1, 2].

**Fig. S2** Negative controls of bacterial growth curves incubated at distinct temperatures (Fig. 3) consisting of LB and R2A media without bacteria.

**Table S1** Bacterial biomass (mg dry weight) recovered from *Paenisporosarcina macmurdoensis* strains B1 and B2, and *Arthrobacter* sp. strains B7 and B8, at their maximum and minimum temperatures for growth. For strains B1 and B2, an additional temperature for each strain (5 ºC for B1 and 10 ºC for B2) was included to ensure that enough biomass for lipid extraction and detection was available. Bacterial biomass was obtained when cultures reached their initial/mid exponential phase and were washed three times with sterile 0.01 M phosphate buffer saline (PBS) to remove nutrients from LB (strains B1 and B2) and R2A (strains B7 and B8) media.

|  |  | Bacterial biomass (mg dw) recovered per temperature | | | | |
| --- | --- | --- | --- | --- | --- | --- |
|  |  | 0 ºC | 5 ºC | 10 ºC | 20 ºC | 25 ºC |
| Bacterial strains | B1 | 32 | 156 | - | 349 | - |
|  | B2 | - | 20 | 223 | 283 | - |
|  | B7 | - | - | 113 | - | 183 |
|  | B8 | - | 186 | - | - | 179 |

**Table S2** Taxonomic ascription of the isolated bacterial strains (B1-B12) using the EzBioCloud database (https://www.ezbiocloud.net/) with a similarity percentage of > 99 %.

| **Isolated strain** | **Hit taxon name and similarity (%) against EzBioCloud database** |
| --- | --- |
| B1 | *Paenisporosarcina macmurdoensis* CMS 21w (99.92 %) |
| B2 | *Paenisporosarcina macmurdoensis* CMS 21w (99.93 %) |
| B3 | *Paenisporosarcina macmurdoensis* CMS 21w (99.93 %) |
| B4 | *Paenisporosarcina macmurdoensis* CMS 21w (99.93 %) |
| B5 | *Paenisporosarcina macmurdoensis* CMS 21w (99.93 %) |
| B6 | *Paenisporosarcina macmurdoensis* CMS 21w (99.92 %) |
| B7 | *Arthrobacter tumbae* LMG 19501 (99.61 %), *Arthrobacter subterraneus* CH7 (99.46 %), *Arthrobacter tecti* LMG 22282 (99.31 %), *Arthrobacter parietis* LMG 22281 (99.23 %) |
| B8 | *Arthrobacter tumbae* LMG 19501 (99.76 %), *Arthrobacter parietis* LMG 22281 (99.69 %), *Arthrobacter tecti* LMG 22282 (99.45 %), *Arthrobacter subterraneus* CH7 (99.37 %) |
| B9 | *Arthrobacter tumbae* LMG 19501 (99.73 %), *Arthrobacter parietis* LMG 22281 (99.64 %), *Arthrobacter tecti* LMG 22282 (99.37 %), *Arthrobacter subterraneus* CH7 (99.27 %) |
| B10 | *Arthrobacter tumbae* LMG 19501 (99.62 %), *Arthrobacter subterraneus* CH7 (99.46 %), *Arthrobacter tecti* LMG 22282 (99.31 %), *Arthrobacter parietis* LMG 22281 (99.23 %) |
| B11 | *Arthrobacter ruber* MDB1-42 (99.77 %), *Arthrobacter agilis* DSM 20550 (99.62 %), *Arthrobacter bussei* KR32 (99.62 %) |
| B12 | *Arthrobacter ruber* MDB1-42 (99.77 %), *Arthrobacter agilis* DSM 20550 (99.62 %), *Arthrobacter bussei* KR32 (99.62 %) |

**Table S3** Results of the Permutational Multivariate Analysis of Variance (PERMANOVA) examining the effects of temperature and bacterial taxonomy (*Paenisporosarcina macmurdoensis* and *Artrhobacter* sp.) on lipid composition. Table shows the degrees of freedom (Df), the sum of squares (SumOfSqs), the proportion of variance explained (R²), the pseudo-F statistic (F), and the *p*-value (*p*). NA indicates Not Applicable.

|  | **Df** | **SumOfSqs** | **R^2^** | **F** | ***p*** |
| --- | --- | --- | --- | --- | --- |
| **Temperature** | 1 | 0.1926446 | 0.1759426 | 4.130317 | 0.039 |
| **Taxonomy** | 1 | 0.5757929 | 0.5258725 | 12.345047 | 0.004 |
| **Residual** | 7 | 0.3264913 | 0.2981849 | NA | NA |
| **Total** | 9 | 1.0949288 | 1.0000000 | NA | NA |

**Table S4** Fatty acid concentrations (µg · g^-1^ dw) as a function of temperature in *Paenisporosarcina macmurdoensis* strains B1 and B2, and *Arthrobacter* sp. strains B7 and B8. Bacterial strains were incubated at their maximum and minimum temperatures for growth. For strains B1 and B2, an additional temperature for each strain (5 ºC for B1 and 10 ºC for B2) was included to ensure that enough biomass for lipid detection was available. Roman numbers in brackets refer to different isomers of the same compound. “DiMe” or “Mme” indicate dimethylated or monomethylated congeners, and “n.d.” stands for non-detected compound.

| **Fatty acids**  **(µg · g^-1^ dw)** | ***Paenisporosarcina macmurdoensis*** | | | | | | |  | ***Arthrobacter* sp.** | | | | | **Neg.**  **control** |
| --- | --- | --- | --- | --- | --- | --- | --- | --- | --- | --- | --- | --- | --- | --- |
|  | **B1** | | |  | **B2** | | |  | **B7** | |  | **B8** | |  |
|  | **0 ºC** | **5 ºC** | **20 ºC** |  | **5 ºC** | **10 ºC** | **20 ºC** |  | **10 ºC** | **25 ºC** |  | **5 ºC** | **25 ºC** |  |
| C_10:0_ | n.d. | n.d. | 6 |  | n.d. | n.d. | n.d. |  | n.d. | n.d. |  | n.d. | n.d. | n.d. |
| C_12:0_ | n.d. | n.d. | 8 |  | 31 | 35 | 21 |  | n.d. | n.d. |  | n.d. | n.d. | n.d. |
| C_13:0_ | n.d. | n.d. | 7 |  | n.d. | n.d. | n.d. |  | n.d. | n.d. |  | n.d. | n.d. | n.d. |
| C_14:0_ | 114 | 93 | 102 |  | 104 | 238 | 182 |  | 133 | 96 |  | 99 | 112 | n.d. |
| C_15:0_ | 39 | 65 | 238 |  | 51 | 222 | 197 |  | 59 | 31 |  | 61 | 36 | n.d. |
| C_16:0_ | 469 | 201 | 221 |  | 394 | 401 | 307 |  | 922 | 600 |  | 646 | 520 | 9 |
| C_17:0_ | n.d. | 17 | 27 |  | 25 | 35 | 16 |  | 31 | 14 |  | 19 | 16 | n.d. |
| C_18:0_ | 109 | 46 | 59 |  | 101 | 66 | 60 |  | 244 | 214 |  | 249 | 219 | 4 |
| C_14:1_ | n.d. | n.d. | n.d. |  | n.d. | n.d. | n.d. |  | 535 | 57 |  | 781 | 198 | n.d. |
| C_14:1ω2_ | n.d. | n.d. | n.d. |  | n.d. | n.d. | n.d. |  | 11 | n.d. |  | n.d. | n.d. | n.d. |
| C_14:1ω5_ | n.d. | n.d. | n.d. |  | n.d. | n.d. | n.d. |  | n.d. | n.d. |  | n.d. | n.d. | n.d. |
| C_16:1_ | 1921 | 837 | 834 |  | 272 | 1447 | 1016 |  | n.d. | n.d. |  | n.d. | n.d. | n.d. |
| C_16:1ω7_ (I) | 663 | 314 | 169 |  | 138 | 573 | 304 |  | 615 | 578 |  | 572 | 520 | n.d. |
| C_16:1ω7_ (II) | 95 | 22 | 22 |  | 52 | 59 | 35 |  | n.d. | n.d. |  | n.d. | n.d. | n.d. |
| C_16:1ω7_ (III) | 46 | 11 | 13 |  | 7 | 21 | 13 |  | n.d. | n.d. |  | n.d. | n.d. | n.d. |
| C_16:1ω7_ (IV) | n.d. | 9 | 11 |  | 34 | 22 | 14 |  | n.d. | n.d. |  | n.d. | n.d. | n.d. |
| C_17:1ω7_ (I) | n.d. | n.d. | 49 |  | 0 | 136 | 89 |  | n.d. | n.d. |  | n.d. | n.d. | n.d. |
| C_17:1ω7_ (II) | 492 | 437 | 601 |  | 104 | 1135 | 795 |  | n.d. | n.d. |  | n.d. | n.d. | n.d. |
| C_17:1ω7_ (III) | n.d. | n.d. | 51 |  | n.d. | 55 | 28 |  | n.d. | n.d. |  | n.d. | n.d. | n.d. |
| C_18:2ω6,9_ | n.d. | n.d. | 20 |  | 13 | 25 | n.d. |  | n.d. | n.d. |  | n.d. | 45 | n.d. |
| C_18:1ω6_ | 147 | 49 | 63 |  | 85 | 91 | 19 |  | 68 | 78 |  | 107 | 108 | n.d. |
| *i*C_14:0_ | 1629 | 483 | 704 |  | 265 | 854 | 625 |  | 65 | 132 |  | 15 | 58 | n.d. |
| *i*C_15:0_ | 140 | 74 | 466 |  | 142 | 540 | 328 |  | 635 | 431 |  | 272 | 867 | n.d. |
| *a*C_15:0_ | 1537 | 782 | 2434 |  | 495 | 2498 | 2392 |  | 2568 | 2444 |  | 2330 | 2602 | n.d. |
| *i*C_16:0_ | 2002 | 590 | 1052 |  | 446 | 974 | 908 |  | 478 | 842 |  | 145 | 362 | n.d. |
| *i*C_17:0_ | n.d. | 11 | 183 |  | 36 | 118 | 115 |  | 458 | 358 |  | 118 | 437 | n.d. |
| *a*C_17:0_ | 500 | 439 | 921 |  | 438 | 908 | 1043 |  | 1684 | 1657 |  | 1509 | 1634 | n.d. |
| *i*C_18:0_ | n.d. | n.d. | 31 |  | n.d. | n.d. | 20 |  | 18 | 56 |  | n.d. | 16 | n.d. |
| *i*C_19:0_ | n.d. | n.d. | 4 |  | n.d. | n.d. | n.d. |  | n.d. | 79 |  | n.d. | 40 | n.d. |
| Mme-C_14:1_ | n.d. | n.d. | n.d. |  | n.d. | n.d. | n.d. |  | 169 | 23 |  | 100 | 82 | n.d. |
| Mme-C_15:1_ | n.d. | n.d. | n.d. |  | n.d. | n.d. | n.d. |  | 172 | 311 |  | 68 | 233 | n.d. |
| Mme-C_16:1_ (I) | n.d. | n.d. | n.d. |  | n.d. | n.d. | n.d. |  | 190 | 286 |  | 94 | 415 | n.d. |
| Mme-C_16:1_ (II) | n.d. | n.d. | n.d. |  | n.d. | n.d. | n.d. |  | 801 | 951 |  | 862 | 1409 | n.d. |
| DiMe-C_9:0_ | n.d. | n.d. | 6 |  | n.d. | n.d. | n.d. |  | n.d. | n.d. |  | n.d. | n.d. | n.d. |
| Mme-C_10:0_ | n.d. | n.d. | 7 |  | n.d. | n.d. | n.d. |  | n.d. | n.d. |  | n.d. | n.d. | n.d. |
| Mme-C_11:0_ | n.d. | n.d. | 10 |  | n.d. | n.d. | n.d. |  | n.d. | n.d. |  | n.d. | n.d. | n.d. |
| Mme-C_12:0_ (I) | n.d. | n.d. | 20 |  | n.d. | n.d. | n.d. |  | n.d. | n.d. |  | n.d. | n.d. | n.d. |
| Mme-C_12:0_ (II) | n.d. | n.d. | 21 |  | n.d. | n.d. | n.d. |  | n.d. | n.d. |  | n.d. | n.d. | n.d. |
| Mme-C_12:0_ (III) | n.d. | n.d. | 69 |  | 12 | 39 | 38 |  | n.d. | n.d. |  | n.d. | n.d. | n.d. |
| Mme-C_14:0_ (I) | n.d. | n.d. | 32 |  | n.d. | 10 | n.d. |  | n.d. | n.d. |  | n.d. | n.d. | n.d. |
| Mme-C_14:0_ (II) | n.d. | n.d. | 27 |  | n.d. | n.d. | n.d. |  | n.d. | n.d. |  | n.d. | n.d. | n.d. |
| Mme-C_14:0_ (III) | n.d. | n.d. | 69 |  | n.d. | n.d. | n.d. |  | n.d. | n.d. |  | n.d. | n.d. | n.d. |
| Mme-C_16:0_ (I) | n.d. | n.d. | 25 |  | n.d. | n.d. | n.d. |  | n.d. | n.d. |  | n.d. | n.d. | n.d. |
| Mme-C_16:0_ (II) | n.d. | n.d. | 38 |  | n.d. | n.d. | n.d. |  | n.d. | n.d. |  | n.d. | n.d. | n.d. |
| Mme-C_18:0_ | n.d. | n.d. | 17 |  | n.d. | n.d. | 15 |  | n.d. | n.d. |  | n.d. | n.d. | n.d. |

**Table S5** Hydrocarbon concentrations (µg · g^-1^ dw) as a function of temperature in *Paenisporosarcina macmurdoensis* strains B1 and B2, and *Arthrobacter* sp. strains B7 and B8. Bacterial strains were incubated at their maximum and minimum temperatures for growth. For strains B1 and B2, an additional temperature for each strain (5 ºC for B1 and 10 ºC for B2) was included to ensure that enough biomass for lipid detection was available. Roman numbers in brackets refer to different isomers of the same compound. “Squalene-der.” stands for squalene-related compounds (i.e., derivative), and “n.d.”, for non-detected compound.

| **Hydrocarbons**  **(µg · g^-1^ dw)** | ***Paenisporosarcina macmurdoensis*** | | | | | | |  | ***Arthrobacter* sp.** | | | | | **Neg.**  **control** |
| --- | --- | --- | --- | --- | --- | --- | --- | --- | --- | --- | --- | --- | --- | --- |
|  | **B1** | | |  | **B2** | | |  | **B7** | |  | **B8** | |  |
|  | **0 ºC** | **5 ºC** | **20 ºC** |  | **5 ºC** | **10 ºC** | **20 ºC** |  | **10 ºC** | **25 ºC** |  | **5 ºC** | **25 ºC** |  |
| C_27:1_ (I) | n.d. | n.d. | n.d. |  | n.d. | n.d. | n.d. |  | n.d. | 29 |  | n.d. | 7 | n.d. |
| C_27:1_ (II) | n.d. | n.d. | n.d. |  | n.d. | n.d. | n.d. |  | 14 | 32 |  | 32 | 36 | n.d. |
| C_28:1_ (I) | n.d. | n.d. | n.d. |  | n.d. | n.d. | n.d. |  | n.d. | n.d. |  | n.d. | 5 | n.d. |
| C_28:1_ (II) | n.d. | n.d. | n.d. |  | n.d. | n.d. | n.d. |  | 30 | 192 |  | 16 | 33 | n.d. |
| C_28:1_ (III) | n.d. | n.d. | n.d. |  | n.d. | n.d. | n.d. |  | n.d. | n.d. |  | n.d. | 12 | n.d. |
| C_28:1_ (IV) | n.d. | n.d. | n.d. |  | n.d. | n.d. | n.d. |  | 71 | 112 |  | 102 | 64 | n.d. |
| C_29:1_ (I) | n.d. | n.d. | n.d. |  | n.d. | n.d. | n.d. |  | n.d. | n.d. |  | n.d. | 38 | n.d. |
| C_29:1_ (II) | n.d. | n.d. | n.d. |  | n.d. | n.d. | n.d. |  | n.d. | 78 |  | n.d. | 102 | n.d. |
| C_29:1_ (III) | n.d. | n.d. | n.d. |  | n.d. | n.d. | n.d. |  | 275 | 361 |  | 227 | 513 | n.d. |
| C_29:1_ (IV) | n.d. | n.d. | n.d. |  | n.d. | n.d. | n.d. |  | 635 | 918 |  | 1116 | 950 | n.d. |
| C_29:1_ (V) | n.d. | n.d. | n.d. |  | n.d. | n.d. | n.d. |  | n.d. | 62 |  | 31 | 17 | n.d. |
| C_30:1_ (I) | n.d. | n.d. | n.d. |  | n.d. | n.d. | n.d. |  | n.d. | n.d. |  | n.d. | 7 | n.d. |
| C_30:1_ (II) | n.d. | n.d. | n.d. |  | n.d. | n.d. | n.d. |  | 37 | 164 |  | 30 | 52 | n.d. |
| C_30:1_ (III) | n.d. | n.d. | n.d. |  | n.d. | n.d. | n.d. |  | 51 | 72 |  | 25 | 7 | n.d. |
| C_30:1_ (IV) | n.d. | n.d. | n.d. |  | n.d. | n.d. | n.d. |  | 296 | 142 |  | 370 | 47 | n.d. |
| C_30:1_ (V) | n.d. | n.d. | n.d. |  | n.d. | n.d. | n.d. |  | n.d. | 172 |  | n.d. | 252 | n.d. |
| C_31:1_ (I) | n.d. | n.d. | n.d. |  | n.d. | n.d. | n.d. |  | n.d. | n.d. |  | n.d. | 7 | n.d. |
| C_31:1_ (II) | n.d. | n.d. | n.d. |  | n.d. | n.d. | n.d. |  | n.d. | n.d. |  | 22 | 43 | n.d. |
| C_31:1_ (III) | n.d. | n.d. | n.d. |  | n.d. | n.d. | n.d. |  | 44 | 74 |  | 114 | 134 | n.d. |
| Squalene-der. (I) | n.d. | n.d. | 15 |  | n.d. | n.d. | n.d. |  | n.d. | n.d. |  | n.d. | n.d. | n.d. |
| Squalene-der. (II) | n.d. | n.d. | 47 |  | 155 | 70 | 46 |  | n.d. | n.d. |  | n.d. | n.d. | n.d. |
| Squalene-der. (III) | n.d. | 72 | 59 |  | n.d. | 103 | 73 |  | n.d. | n.d. |  | n.d. | n.d. | n.d. |
| Squalene-der. (IV) | 31 | 60 | 45 |  | n.d. | 57 | 41 |  | n.d. | n.d. |  | n.d. | n.d. | n.d. |
| Squalene-der. (V) | 1067 | 995 | 657 |  | 336 | 1580 | 1162 |  | n.d. | n.d. |  | n.d. | n.d. | n.d. |
| Squalene-der. (VI) | n.d. | n.d. | 7 |  | n.d. | n.d. | n.d. |  | n.d. | n.d. |  | n.d. | n.d. | n.d. |
| Squalene-der. (VII) | n.d. | n.d. | 8 |  | n.d. | n.d. | n.d. |  | n.d. | n.d. |  | n.d. | n.d. | n.d. |
| Squalene-der. (VIII) | n.d. | n.d. | 18 |  | n.d. | n.d. | n.d. |  | n.d. | n.d. |  | n.d. | n.d. | n.d. |

**Table S6** Alcohol concentrations (µg · g^-1^ dw) as a function of temperature in *Paenisporosarcina macmurdoensis* strains B1 and B2, and *Arthrobacter* sp. strains B7 and B8. Bacterial strains were incubated at their maximum and minimum temperatures for growth. For strains B1 and B2, an additional temperature for each strain (5 ºC for B1 and 10 ºC for B2) was included to ensure that enough biomass for lipid detection was available. Roman numbers in brackets refer to different isomers of the same compound. “Isoprenoid alc.” stands for isoprenoidal alcohol, and “n.d.”, for non-detected compound.

| **Alcohols**  **(µg · g^-1^ dw)** | ***Paenisporosarcina macmurdoensis*** | | | | | | |  | ***Arthrobacter* sp.** | | | | | **Neg.**  **control** | |
| --- | --- | --- | --- | --- | --- | --- | --- | --- | --- | --- | --- | --- | --- | --- | --- |
|  | **B1** | | |  | **B2** | | |  | **B7** | |  | **B8** | |  |  |
|  | **0 ºC** | **5 ºC** | **20 ºC** |  | **5 ºC** | **10 ºC** | **20 ºC** |  | **10 ºC** | **25 ºC** |  | **5 ºC** | **25 ºC** | |  |
| 2-C_15:0_ | 3 | 3 | 11 |  | n.d. | 4 | 5 |  | n.d. | n.d. |  | n.d. | n.d. | | n.d. |
| 1-C_16:0_ | 81 | 11 | 22 |  | 18 | 30 | 30 |  | 28 | 25 |  | 20 | 20 | | n.d. |
| 1-C_18:0_ | 43 | 6 | 18 |  | 24 | 11 | 16 |  | 12 | 10 |  | 6 | 7 | | n.d. |
| 1-C_20:0_ | 6 | 1 | n.d. |  | 9 | 1 | 2 |  | 3 | 2 |  | 2 | 1 | | n.d. |
| 1-C_22:0_ | 3 | 1 | n.d. |  | 1 | 1 | 1 |  | 1 | 1 |  | 1 | 6 | | n.d. |
| Farnesol | 1 | 1 | 2 |  | n.d. | 3 | 2 |  | 1 | n.d. |  | n.d. | n.d. | | n.d. |
| Isoprenoid alc. (I) | 19 | 10 | 22 |  | 1 | 25 | 20 |  | n.d. | n.d. |  | n.d. | n.d. | | n.d. |
| Isoprenoid alc. (II) | 26 | 13 | 117 |  | 8 | 33 | 28 |  | n.d. | n.d. |  | n.d. | n.d. | | n.d. |
| Isoprenoid alc. (III) | 177 | 73 | 51 |  | 3 | 155 | 127 |  | n.d. | n.d. |  | n.d. | n.d. | | n.d. |
| Isoprenoid alc. (IV) | 63 | 33 | 152 |  | 11 | 187 | 150 |  | n.d. | n.d. |  | n.d. | n.d. | | n.d. |
| Isoprenoid alc. (V) | n.d. | n.d. | n.d. |  | n.d. | 70 | 53 |  | n.d. | n.d. |  | n.d. | n.d. | | n.d. |

**Table S7** Relative abundance of genera within the dominant Actinomycetota and Bacillota phyla in the ancient and desiccated Antarctic microbial mat. This information is extracted from the massive 16S rRNA gene analysis conducted by Lezcano et al. (2022) [1]. When the genus-level classification was not possible, the relative abundance of the next highest taxonomic rank is provided, followed by "unclassified". *Arthrobacter* and *Paenisporosarcina* are highlighted in bold.

| **Phylum** | **Genus (or the higher taxonomic rank)** | **Relative abundance (%)** |
| --- | --- | --- |
| Actinomycetota | Acidimicrobiales_unclassified | 0.47 |
|  | *Aciditerrimonas* | 0.01 |
|  | Actinobacteria_unclassified | 0.79 |
|  | Actinomycetales_unclassified | 1.67 |
|  | *Actinotalea* | 0.31 |
|  | *Aeromicrobium* | 1.80 |
|  | *Aquihabitans* | 0.27 |
|  | ***Arthrobacter*** | **0.11** |
|  | *Conexibacter* | 0.65 |
|  | Coriobacteriaceae_unclassified | 0.34 |
|  | *Corynebacterium* | 0.00 |
|  | *Cryobacterium* | 1.04 |
|  | *Demequina* | 0.20 |
|  | *Dermacoccus* | 0.00 |
|  | *Euzebya* | 0.00 |
|  | *Gaiella* | 0.03 |
|  | Geodermatophilaceae_unclassified | 0.00 |
|  | *Lamia* | 0.01 |
|  | Lamiaceae_unclassified | 0.70 |
|  | *Ilumatobacter* | 0.32 |
|  | Intrasporangiaceae_unclassified | 0.01 |
|  | *Marmoricola* | 0.18 |
|  | *Micromonospora* | 1.74 |
|  | *Nitriliruptor* | 0.00 |
|  | *Nocardioides* | 3.78 |
|  | Propionibacteriaceae_unclassified | 0.34 |
|  | Solirubrobacterales_unclassified | 0.18 |
|  | *Streptomyces* | 0.12 |
|  | *Tetrasphaera* | 2.24 |
| Bacillota | *Acetoanaerobium* | 0.15 |
|  | *Acetobacterium* | 0.04 |
|  | *Alkaliphilus* | 0.91 |
|  | *Anaerovorax* | 0.06 |
|  | Bacillales_unclassified | 0.70 |
|  | Bacilli_unclassified | 0.04 |
|  | *Cellulosilyticum* | 2.67 |
|  | Clostridia_unclassified | 0.03 |
|  | Clostridiaceae_1_unclassified | 0.23 |
|  | Clostridiaceae_2_unclassified | 0.58 |
|  | Clostridiales_unclassified | 12.77 |
|  | *Clostridium_III* | 1.83 |
|  | *Clostridium_sensu_stricto* | 13.12 |
|  | *Clostridium_XlVa* | 0.45 |
|  | *Dehalobacter* | 0.00 |
|  | *Desulfosporosinus* | 26.07 |
|  | *Desulfuribacillus* | 0.32 |
|  | *Dethiobacter* | 0.09 |
|  | Eubacteriaceae_unclassified | 0.04 |
|  | Firmicutes_unclassified | 0.91 |
|  | *Gracilibacter* | 0.02 |
|  | Lachnospiraceae_unclassified | 0.06 |
|  | Natranaerovirga_unclassified | 0.10 |
|  | *Oxobacter* | 0.01 |
|  | Paenibacillaceae_1_unclassified | 0.05 |
|  | *Paenibacillus* | 0.20 |
|  | ***Paenisporosarcina*** | **0.07** |
|  | Peptococcaceae_1_unclassified | 0.14 |
|  | *Planococcus* | 0.06 |
|  | *Planomicrobium* | 0.04 |
|  | *Proteinivorax* | 0.03 |
|  | *Psychrosinus* | 0.13 |
|  | Ruminococcaceae_unclassified | 2.03 |
|  | *Saccharofermentans* | 0.00 |
|  | *Sedimentibacter* | 0.03 |
|  | *Staphylococcus* | 0.00 |
|  | *Syntrophomonas* | 0.01 |
|  | *Tissierella* | 1.23 |
|  | Veillonellaceae_unclassified | 2.36 |
|  | *Youngiibacter* | 0.47 |
| Other phyla |  | 14.61 |

**References**

1.	Lezcano MÁ, Sánchez-García L, Quesada A, et al (2022) Comprehensive Metabolic and Taxonomic Reconstruction of an Ancient Microbial Mat From the McMurdo Ice Shelf (Antarctica) by Integrating Genetic, Metaproteomic and Lipid Biomarker Analyses. Front Microbiol 13:799360. https://doi.org/10.3389/fmicb.2022.799360

2.	Blanco Y, Gallardo-Carreño I, Ruiz-Bermejo M, et al (2017) Critical Assessment of Analytical Techniques in the Search for Biomarkers on Mars: A Mummified Microbial Mat from Antarctica as a Best-Case Scenario. Astrobiology 17:984–996. https://doi.org/10.1089/ast.2016.1467
